# Morphological and ecological adaptation of limpet-shaped top shells (Gastropoda: Trochidae: Fossarininae) to wave-swept rock reef habitats

**DOI:** 10.1371/journal.pone.0197719

**Published:** 2018-08-22

**Authors:** Luna Yamamori, Makoto Kato

**Affiliations:** Graduate School of Human and Environmental Studies, Kyoto University, Sakyo, Kyoto, Japan; University of California, UNITED STATES

## Abstract

Flattening of coiled shells has occurred in several gastropod lineages, while the evolutionary process of shell flattening is little known. The subfamily Fossarininae of the top shell family (Trochidae) is unique, because it includes four genera at various stages of shell flattening. *Broderipia* and *Roya*, have zygomorphic shells that has lost coiling, while the sister genera, *Fossarina* and *Synaptocochlea*, have respectively turbiniform and auriform shells. Therefore, comparisons of biology, habitats and detailed morphology among these four genera may help us to detect selection pressure driving shell flattening and loss of coiling. Although *Broderipia* has recently been identified as living symbiotically in the pits of sea urchins, the habitats and biology of the other three Fossarininae species, especially *Roya* are poorly known. After an extensive search on rocky shores of the Japanese Archipelago, we found live *Roya eximia* snails on intertidal/subtidal rock surfaces exposed to strong waves. *Roya* snails crept on the bare rock surface to graze periphyton at low tide, and fled into vacant barnacle shells at high tide. Comparison of the morphology of soft bodies in Fossarininae revealed that the columellar muscle of flattened species has been drastically elongated and arranged in posterior semi-outer edge of the flattened shell as observed in true limpets. The flattering and loss of coiling of the shell in *Roya* caused acquisition of a zygomorphic flat body, retraction of coiled visceral mass, and expansion of the foot sole. All of these changes improved tolerance against strong waves and the ability to cling to rock surfaces, and thus enabled a lifestyle utilizing both wave-swept rock surfaces and the inside of vacant barnacle shells.

## Introduction

Molluscs exhibit a wide range of shell forms as adaptations to surrounding environmental conditions [1], and for defense against predators [2]. In the history of shell-shape evolution, flattening of the coiled shell is one of the most common strategies; some of these flattened shells subsequently lost their coiling, forming limpet-shaped shells. Limpet-shaped shells are adopted in diverse gastropod lineages, e.g., Patellogastropoda, Cocculiniformia, Lepetodriloidea, Fissurellidae, Phenacolepadidae, Hipponicidae, Calyptraeidae, Umbraculidae, Trimusculidae, Siphonariidae, Ancylidae, some Capulidae, *Thyca crystallina* (Eulimidae), *Amathina* (Amathinidae), and a portion of Fossarininae (Trochidae), among other groups [3].

Because limpet-shape is thought to be not well adapted to intense competition and predation, it is thought to be originated in refugial habitats, however, there are some advantages of limpet-shape, depending on their habitats and feeding types [4]. For example, because of the structurally compact body, limpets potentially have large respiratory surface. This large respiratory surface is advantageous not only for oxygen-poor deep sea, but also for suspension feeding as seen in Calyptraeidae [5]. Other major advantages of the limpet-shaped shells are hypothesized to be reduction of the risk to be pulled off by strong waves, improvement of adhering strength, and rapid locomotion, which are enabled by low-conical shell and large foot [3]. Although limpet-shaped shells show various ecologies, the factors that promote the evolution of limpet-shaped shells have not yet been identified, because most members of the aforementioned families have limpet-shaped shells and species in transition from coiled to non-coiled shells are rare.

**Fig 1:**
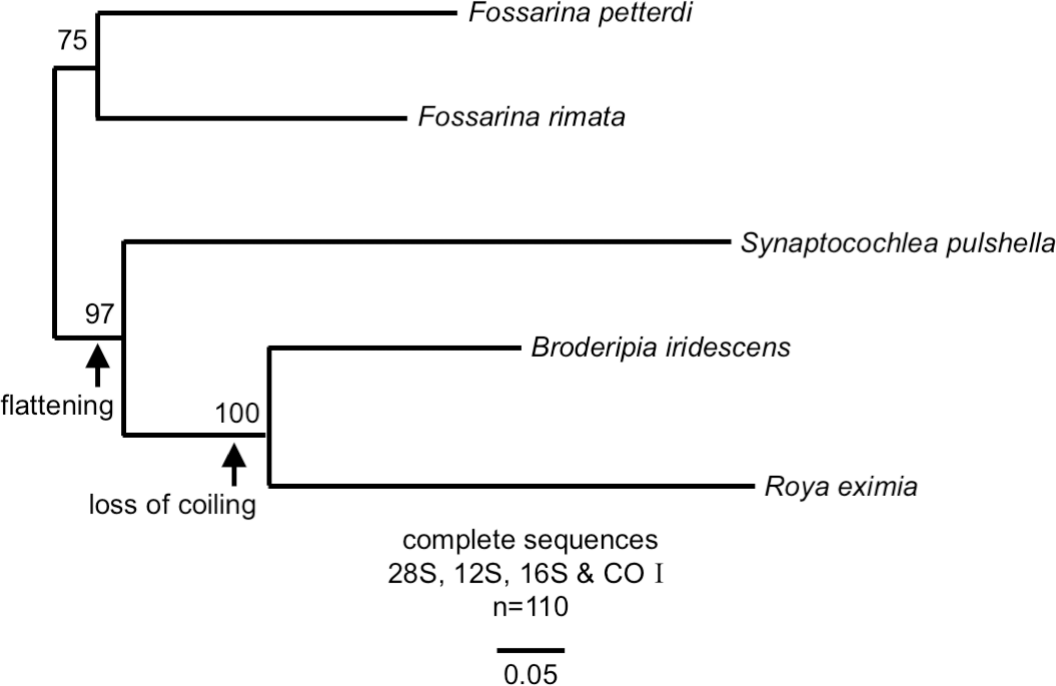
The phylogenetic tree of subfamily Fossarininae (Williams et al. 2010, modified).

The top-shell family Trochidae is characterized by conical, coiled shells and an alga-grazing habit, although some linages contain filter feeders (Umboniinae). In Trochidae, the two subfamilies (Fossarininae and Stomatellinae) contain species with flattened shells. The phylogenetic tree of Fossarininae [6] suggests that shell flattening has occurred from a turbiniform shell to an auriform shell, and subsequently to a cap-shaped shell (Fig 1). Because the four genera of Fossarininae are currently at various stages in the evolutionary process of shell flattening, comparisons of their biology and habitats will help us to detect selection pressure driving shell-flattening and loss of coiling. However, only limited information is available about the ecology of Fossarininae species. *Broderipia*, which has an extremely flat shell, has recently been revealed to be symbiotic in the pits of sea urchins, and its flat limpet-shaped shell is apparently adaptive to life in the narrow open space of the pits [7]. The habitat and biology of other Fossarininae species, especially *Roya* are still poorly known.

To detect selective pressures acting on shell flattening and the loss of coiling in Fossarininae, we first conducted an extensive search for habitats of the key genus, *Roya*. Because *Roya* snails are found in low intertidal areas of wave-swept rocky reefs, we carried out a field survey of the macrobenthic and macrophytic communities on various types of rocky reefs surrounding *Roya* snails, and observed the diurnal behavior of the snails. To determine their feeding biology, radula and gut contents of *Roya* snails were also examined. By superimposing the obtained data on a cladogram of Fossarininae, we discussed the evolution of shell flattening and loss of coiling in gastropods.

## Materials and methods

### Study sites

**Fig 2:**
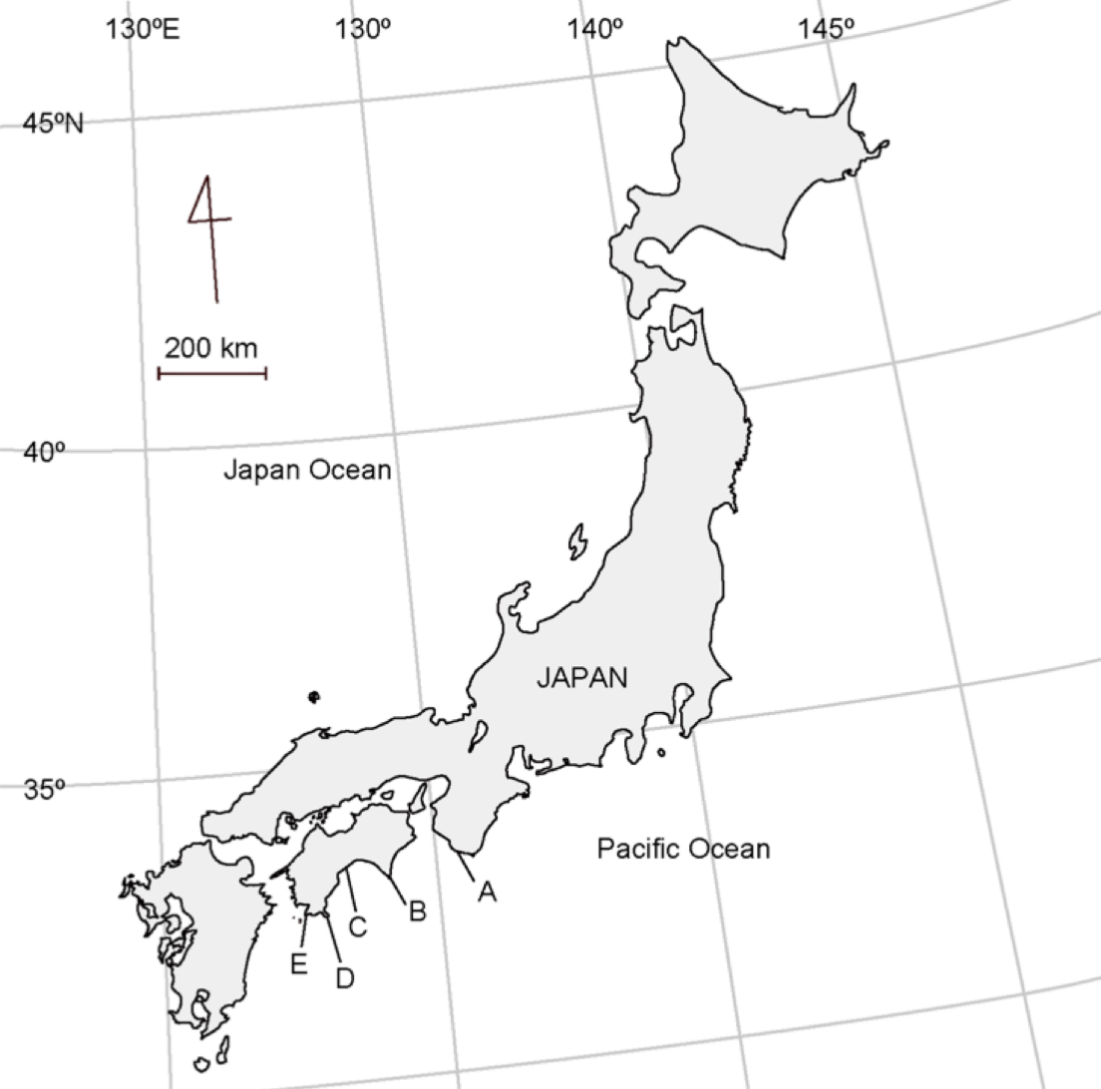
Locations of the study sites. A: Toshima in Shirahama, Wakayama Prefecture, B: Muroto Cape, C: Goshikihama, D: Chihiro Cape, E: Kashiwajima Island in Kochi Prefecture.

**Fig 3:**
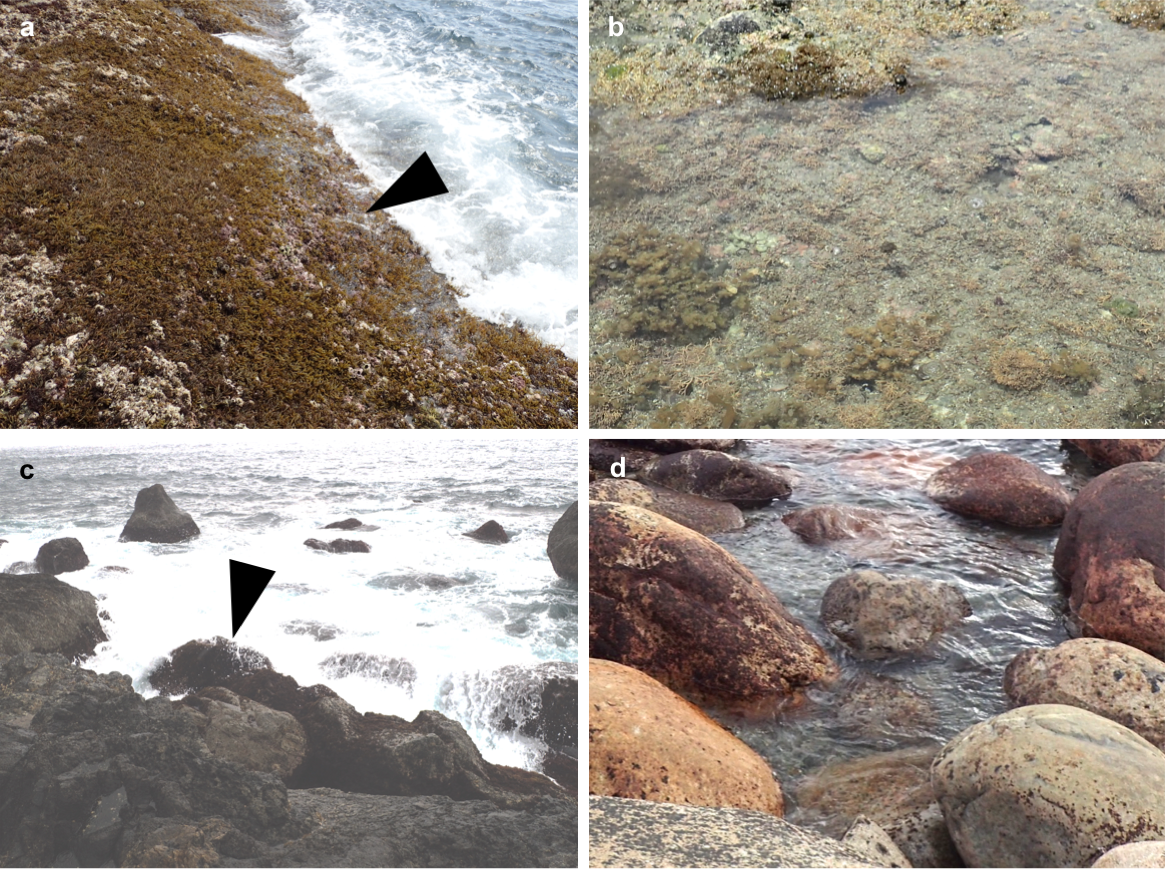
Study sites and habitats of snail species belonging to Fossarininae. a: Exposed reef at site A; arrowhead shows the tidal level inhabited by *R*. *eximia*. b: Protected reef at site A. c: Exposed reef at site E; arrowhead shows the rock inhabited by *R*. *eximia*. d: Protected reef at site E.

This study was conducted on rocky shores in the Japanese Archipelago, which are influenced by the warm Kuroshio Current. Through a preliminary search for the rare limpet-shaped trochid snail *R*. *eximia*, five populations were identified on the southern coasts of the Kii Peninsula and Shikoku Island (sites A–E in Fig 2). No specific permissions were required for these locations, and neither endangered nor protected species were involved in this field study. At all sites, the maximum tidal range during spring tide is around 2.0 m. Because the habitat of *R*. *eximia* is constantly exposed to violent waves, accessible sites were rare. The only accessible coastal sites were sites A (33°69′51′′N, 135°33′58′′E) and E (32°46'00"N, 132°37'18"E). Site A is a small near-shore island called Toshima, the west coast of which faces the Kii Channel and is exposed to strong waves. The rock bed is conglomerate mixed with brittle sandstone, and the surface remains rough due to constant erosion from waves (Fig 3A and 3B). Site E is a rocky and boulder-filled shore of Kashiwajima Island, which is constantly exposed to strong waves (Fig 3C and 3D). The large boulders accumulated on the shore are made up of hard and smooth igneous rock [8].

### Census on macroalgae and macrobenthos

In the lower intertidal zones of sites A and E, two different settings of rock surfaces were chosen, which were exposed to and protected from strong waves. We refer to the former and latter settings as exposed and protected reefs, respectively. At first, we evaluated the intensity of waves by measuring the wave beat frequency and the average/maximum wave height (i.e., vertical range to which a wave dashes up on rock reef) in a minute by checking the video of waves at low tides at each site. In both rock surface settings, 10 quadrats (10 × 10 cm) were set, and all macroalgae and macrobenthos in each quadrat were examined in terms of species, abundance (for macrobenthos) and coverage (for macroalgae) during a spring tide in April 2017.

### Morphology and diet of Fossarininae

The shell, soft body and radula morphologies of the four Fossarininae species were compared. Firstly, the side view of the living snail and side view and aperture view of their shells were photographed. Secondly, to examine the soft bodies, snails were anesthetized in 8% MgCl_2_ solution for 1 h, and fixed in 4% formalin solution for about 12 h. After fixation, snails were removed from the formalin solution, rinsed with flowing fresh water for 30 minutes, and transferred to 70% Ethanol to examine soft bodies under optical microscope. Radulae were removed from each snail and examined using an electron microscope. Lastly, to determine the diet of Fossarininae snails, the stomach and intestinal contents were examined. Fossarininae snails were collected at site A and immediately fixed in 4% formalin solution for 12 h, and rinsed with water. The stomach and intestine of each Fossarininae snail were removed and opened in water; approximately 1/5 of the contents were observed and its organic matters were counted using an optical microscope.

### Diurnal behavior of *R*. *eximia*

At site A, diurnal changes in the distribution of *R*. *eximia* snails were surveyed in the intertidal area at high tide, the middle of the ebb tide (awash time of their habitat tidal level) and low tide in the daytime, and at low tide in the nighttime on 15^th^ April 2017. In each census, *R*. *eximia* snails were sought out not only on the rock surface, but also in the interspaces of sessile organisms and inside vacant shells of barnacles; their behavior was observed.

## Results and discussion

### Rock-surface environment and macrobenthic community at each site

As the wave intensity, frequencies and the average and maximum heights of waves per minutes are shown in Table 1. The wave intensities were higher in exposed than protected rock reefs.

**Table 1:** The wave intensities at protected and exposed rock reefs in site A and E.

		frequency (± sd) (beats / min.)	average (± sd) height in a minute (cm)	maximum (± sd) height in a minute (cm)
site A	protected	22.7 ± 5.6	2.3 ± 1.0	5.5 ± 1.1
	exposed	21.3 ± 6.1	112.4 ± 12.1	223.9 ± 25.5
site E	protected	35.8 ± 4.2	4.9 ± 1.3	8.7 ± 2.1
	exposed	14.4 ±3.3	122.7 ± 21.1	284.9 ± 18.9

The surfaces of the exposed reefs in the lower intertidal zones of sites A and E were covered with 13 species of encrusting and branching algae. The proportions of bare surface, i.e., areas without cover of macroalgae or sessile organisms, of exposed and protected rock surfaces were 8.86% and 47.1% at site A, and 24.3% and 20.8% at site E, respectively. Most rock surfaces were not covered with sand, although the protected rock surfaces of site A were partly covered with sand. This low algal coverage of protected reefs may be caused by sedimentation on rock surfaces, as the growth of branching algae is hindered by sedimentation [9–10].

**Fig 4:**
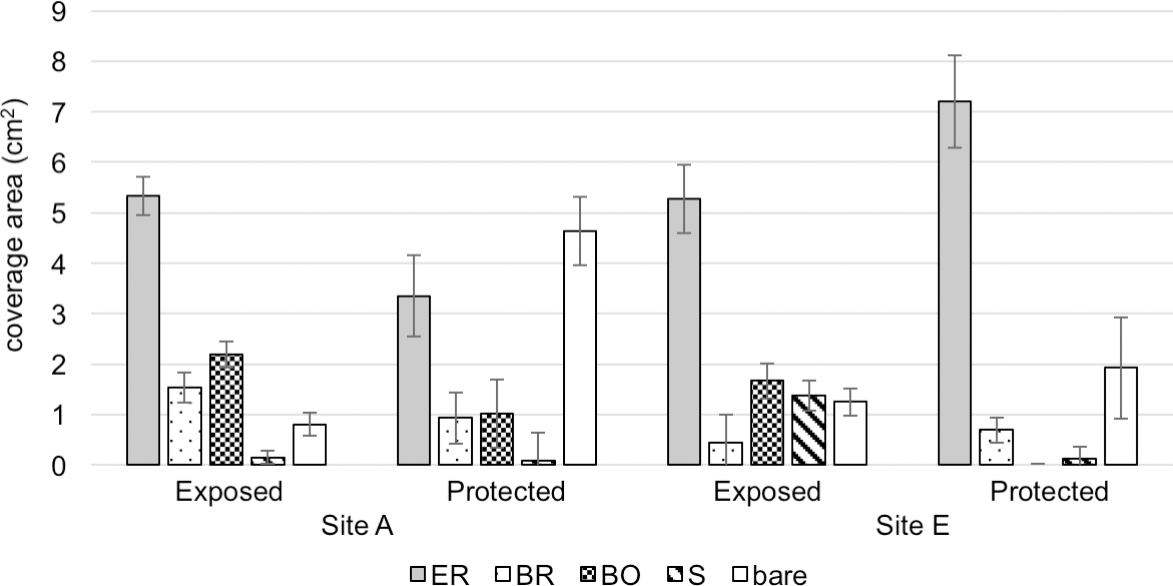
**Mean coverage (cm^2^) of various algae and sessile organisms in 10 × 10 cm quadrats (n = 10) set on exposed and protected rock surfaces at sites A and E**. ER: encrusting Rhodophyta, BR: branching Rhodophyta BO: branching Ochrophyta. S: sessile invertebrates, bare: bare surface.

The most frequent and dominant macroalgae were the encrusting coralline red algae *Lithophyllum* spp. and encrusting non-coralline red alga *Hildenbrandia rubra* (S1 Table). The branched coralline algae *Amphiroa beauvoisii* and *Serraticardia maxima* were found only at site A. The assemblages of brown algae differed between the two sites, as well as between exposed and protected microhabitats. At site A, the rock surfaces exposed to strong waves were dominated by the large brown algae *Sargassum fusiforme* and *S*. *patens*, while more protected rock surfaces were often inhabited by the brown alga *Palisada intermedia*. On the other hand, at site E, exposed rock surfaces were inhabited by *P*. *intermedia* and *Ishige okamurae*, while no brown algae were observed on protected rock surfaces. The coverages of macroalgae and sessile organisms at sites A and E on exposed and protected rock surfaces are shown in Fig 4. The coverage of branching red algae on exposed rock surfaces was greater at site A than site E (t-test, p = 0.011), whereas the coverage of encrusting red algae on protected rock surfaces was greater at site E than site A (t-test, p = 0.0047). Coverages of encrusting red algae and branching brown algae at site A were significantly greater on exposed rock surfaces than on protected rock surfaces (t-test: p = 0.033 for encrusting red algae; p = 0.047 for branching brown algae). In addition to these macroalgae, the rock surfaces of the intertidal zones were covered with sessile organisms, such as barnacles and a sessile vermetid snail, *Serpulorbis imbricatus*. At site A, three large barnacle species, *Megabalanus volcano*, *Tetraclita japonica* and *T*. *squamosa* were observed on exposed rock surfaces, while no barnacles were found on protected rock surfaces. At site E, four barnacle species were observed. Two species of small barnacles, *Fistulobalanus albicostatus* and *Chthamalus challengeri*, inhabited the protected rock surfaces, while three species, including the two large tetraclitid species*T*. *japonica* and *T*. *squamosa*, inhabited exposed rock surfaces (S1 Table). Coverage of sessile organisms on exposed rock surfaces was greater at site E than site A (t-test, p = 0.00050); at site E, coverage was greater on exposed rock surfaces than protected rock surfaces (t-test, p = 0.00032). The availability of plankton is affected by wave action [11], therefore, it is assumed that large barnacles inhabit exposed rock surfaces where plenty of planktons are provided by strong waves.

**Fig 5:**
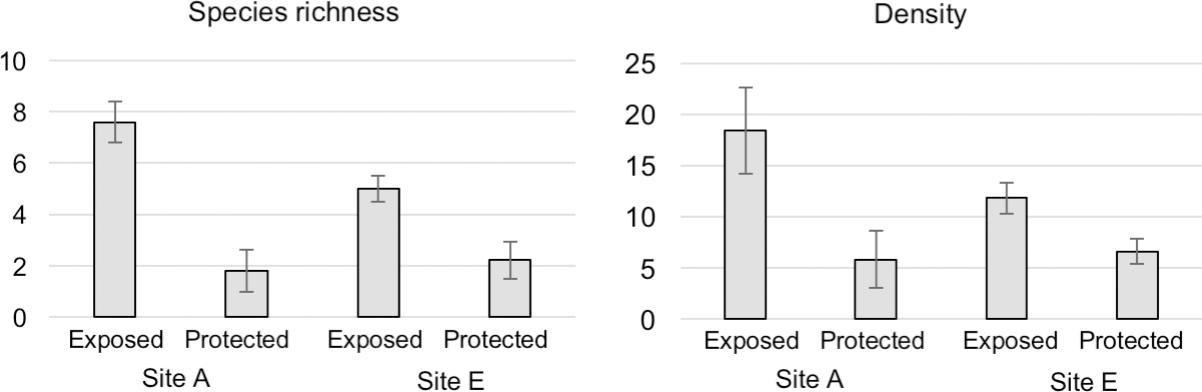
Species richness and density of the macrobenthos in 10 × 10 cm quadrats (n = 10) set on exposed and protected rock surfaces at sites A and E.

The composition of mobile fauna was also influenced by wave strength. From the comparison of species richness and density between exposed and protected reefs (Fig 5), species richness on exposed habitats was higher at site A than at site E (t-test, p = 0.018), and species richness on the exposed reef was higher than on the protected reef at both sites A and E (t-test: p = 0.000011 for site A, p = 0.00040 for site E). The density of macrobenthos on the exposed reef was also greater than that on the protected reef (t-test: p = 0.024 for site A, p = 0.006 for site E).

**Fig 6:**
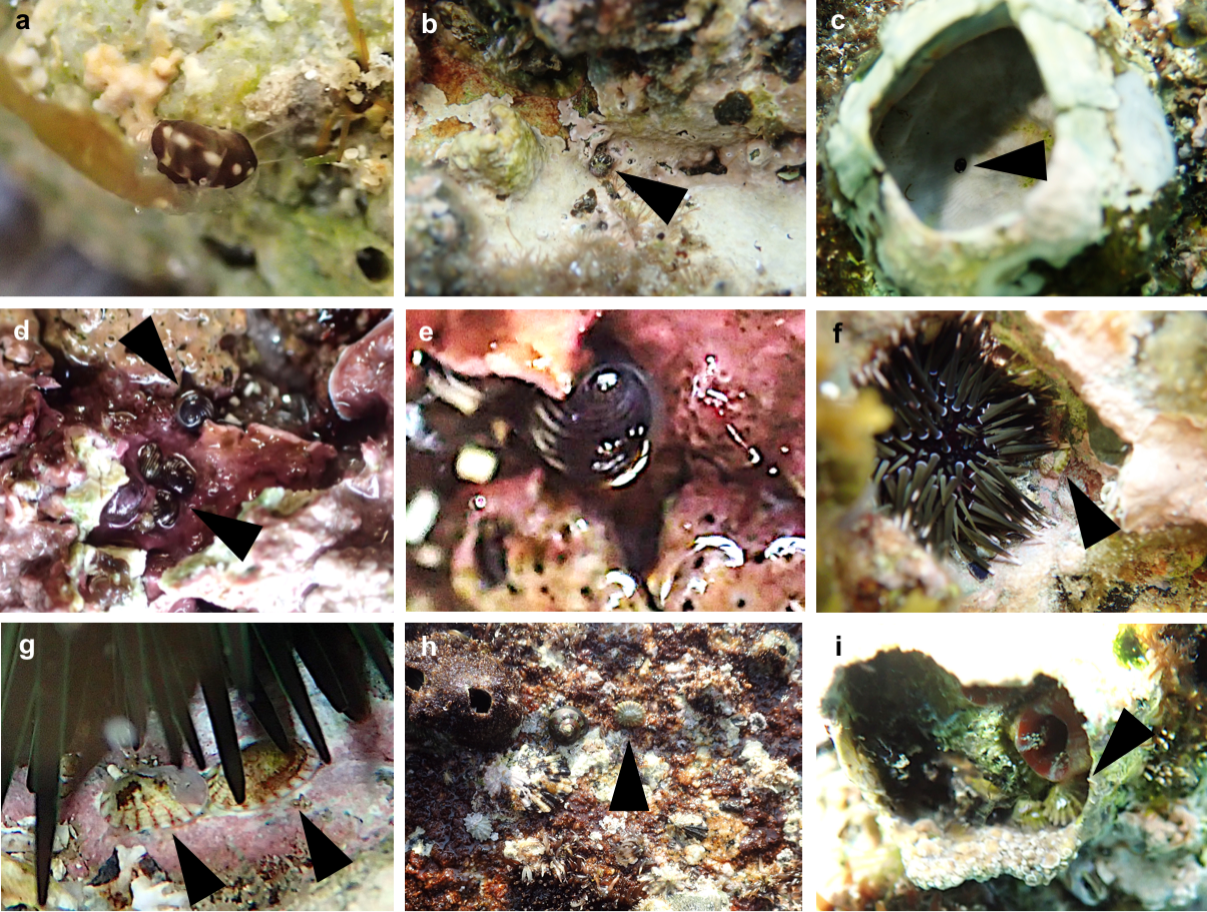
Microhabitats of Fossarininae snails. a−c: *F*. *picta* in rock crevices of protected (a) and exposed (b) rock reefs, and in vacant shells of the barnacle *Megabalanus volcano* on exposed reefs (c); sandy sediments are sometimes accumulated in crevices, as shown in a. d−e: *S*. *pulchella* in a rock crevice. f−g: *B*. *iridescens* in sea urchin pits. h−i: *R*. *eximia* found on wave-swept rock surfaces at low tide (h), and in vacant shells of the barnacle *M*. *volcano* at high tide (i).

At both sites, four Fossarininae species were observed in four microhabitats: open rock surfaces, crevices, sea urchin pits and vacant barnacle shells (Table 2). *Fossarina picta* was found on both protected and exposed reefs, specifically on open rock surfaces of the protected reef (Fig 6A) and inside crevices and vacant shells of barnacles on the exposed reef (Fig 6B and 6C). *Synaptocochlea pulchella* was found in crevices on rocks in the exposed reef (Fig 6D and 6E). *Broderipia iridescens* was observed exclusively inside the pits or crevices inhabited by sea urchins (Fig 6F and 6G). *R*. *eximia* was found on bare rock surfaces around barnacle colonies in exposed area (Fig 6H). The density of *R*. *eximia* at site E was significantly greater than at site A (t-test, p = 0.033).

**Table 2:** The numbers of individuals of the Fossarininae species observed in each habitat at low tide (from 10 quadrats in both site A and E, 200cm^2^ in total).

	Open rock surface	Crevice	Sea urchin's pit	Vacant barnacle shell	Total
*F*. *picta*	6	2	0	3	11
*S*. *pulchella*	0	2	0	0	2
*B*. *iridescens*	0	0	39	0	39
*R*. *eximia*	23	0	0	0	23

It is important to note that *Montfortula picta* is a snail in the coil-less family Fissurellidae (Vetigastropoda), with a shell that bears a remarkable resemblance to that of *R*. *eximia*. *M*. *picta* was observed only on the exposed reef, and not on the protected reef, at both sites A and B, as was also observed for *R*. *eximia*. The coexistence of morphologically similar, phylogenetically distant snail species suggests that the limpet-shaped shell morphology is a product of convergence due to adaptation to life on exposed rock reefs.

### Diurnal behavior of *Roya eximia*

**Fig 7:**
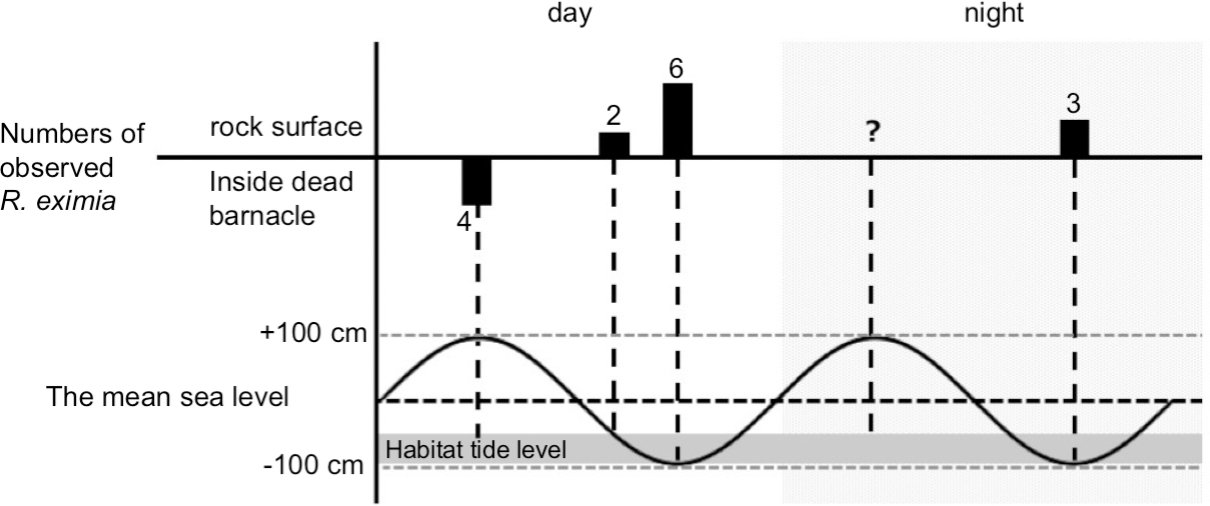
Diurnal changes of tidal level (wavy line) and observed number of *R*. *eximia* snails (solid columns). The habitat of the snail in reference to the tidal level is illustrated as a gray band. *Roya* snails were observed on wet rock surfaces at low tide, while they were found inside vacant barnacle shells on submerged rock surfaces at high tide.

Diurnal changes in the number of *R*. *eximia* snails found at site A are shown in Fig 7. The habitat of *R*. *eximia* was within 50 cm of the ebb tide line. This habitat emerges from the water for about 1 hour during the spring tide, and is constantly splashed during its emergence. During the daytime high tide, four *R*. *eximia* were found inside vacant shells of the barnacle *Megabalanus volcano*, completely covered with water (Fig 6I), and all four snails had tightly adhered their bodies to the inner surface of the barnacle shells, and did not move. At mid-ebb tide in the daytime, on the other hand, the snails swiftly crept over rock surfaces awash with seawater and grazed. When the tide was low during the daytime, six snails were observed on the wet surface of emergent rocks. Two snails slowly and intermittently moved over the emergent zone and grazed, while the other four snails remained motionless, with their bodies tightly adhered to the rock surface. At the nighttime low tide, three snails were observed remaining motionless on the wet rock surface.

This behavioral pattern contrasts with many non-homing limpets that follow the wash zone up and down. This vertical movement is considered to prevent attacks from aquatic predators, and simultaneously to minimize desiccation stress caused by exposure to air [12–13]. In contrast, *Roya* snails minimize predation pressure by moving over wave-swept rock surfaces at mid-low tide and hiding in vacant shells of barnacles at high tide, rather than vertically migrating. Because wave-swept rock surfaces are scarcely inhabited by carnivorous muricid snails, *Roya* snails are largely free from predation. Although submerged rock surfaces are potentially vulnerable to carnivorous fishes at high tide, the vacant shells of barnacles act as refugia from predatory fishes.

### Morphology of the shell and soft body of Fossarininae

**Fig 8:**
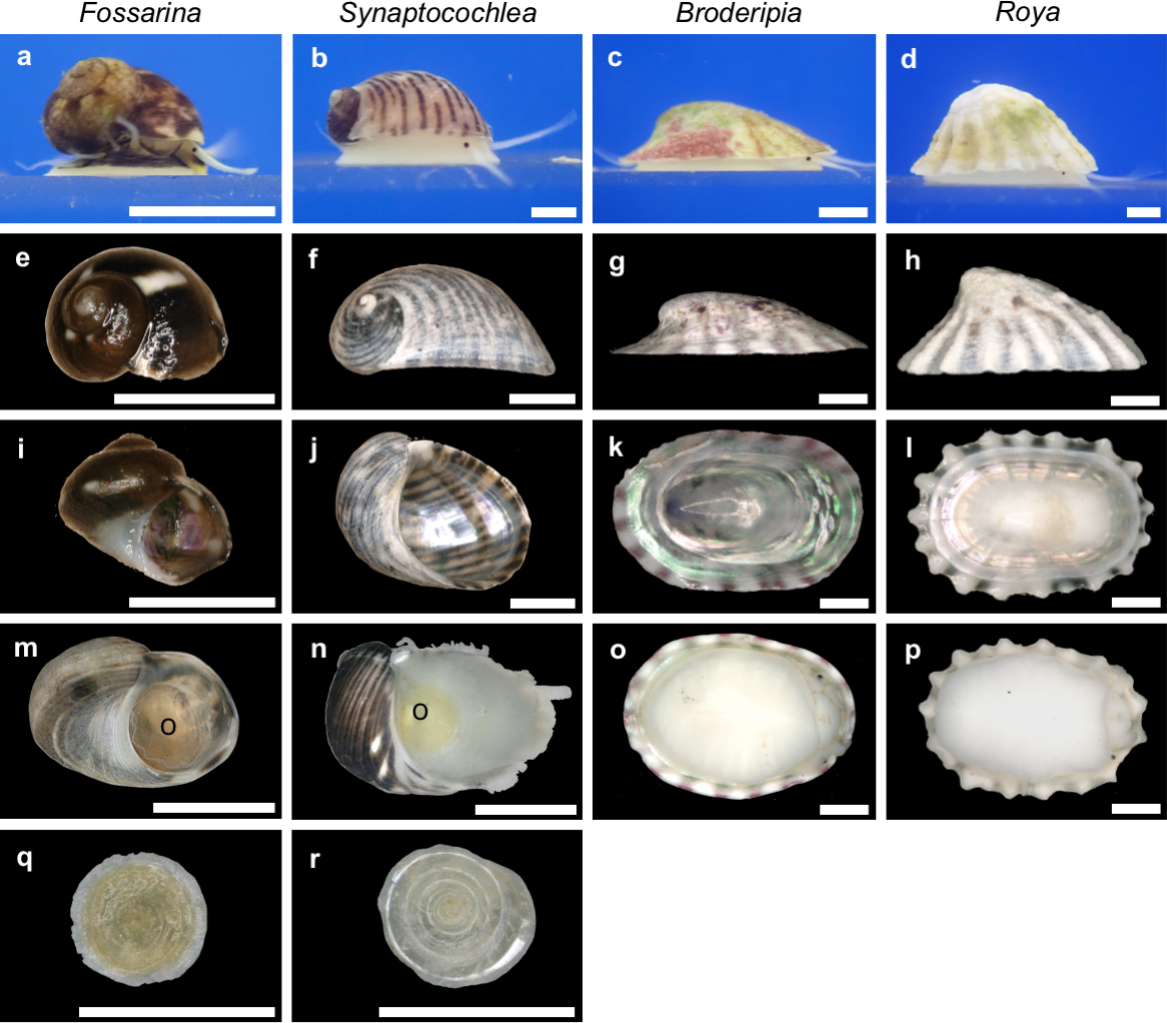
Side and ventral views of living snails and shells of four Fossarininae species, and opercula of *F picta* and *S*. *pulchella*. *F picta* (a, e, i, m, q); *S*. *pulchella* (b, f, j, n, r); *B*. *iridescens* (c, g, k, o); *R*. *eximia* (d, h, l, p). *Scale bar* = 1 mm.

The side views of a living snail, and side and aperture views of shells of four Fossarinae species are shown in Fig 8. *F*. *picta* has a turbiniform shell and four pairs of epipodial tentacles (Fig 8A, 8E and 8I). *S*. *pulchella* has an auriform shell and four pairs of epipodial tentacles (Fig 8B, 8F and 8J). *B*. *iridescens* has a depressed limpet-shaped shell and three pairs of epipodial tentacles (Fig 8C, 8G and 8K). *R*. *eximia* (Fig 8D, 8H and 8L) has a higher limpet-shaped shell armed with radial ribs, and three pairs of epipodial tentacles. The shell morphology of *Roya* is advantageous for survival in wave-swept habitats for the following reasons. First, limpet-shaped shells are more tolerant of strong waves than coiled shells [14]. Second, limpet-shaped shells with large apertures promote the development of a highly contracted soft body with a large foot sole, which would confer strong clinging ability [15, 16]. Third, the radial ribs of the shell (Fig 8H) may reinforce structural integrity, defensive capability against carnivores or light interception. Limpets living in areas exposed to direct sunlight tend to have more ribs and nodules on the shell than those living in shaded areas, which serve to radiate heat [17], while shell ornamentation, including spines and ribs, offers protection against drilling carnivores [18–19]. The first and second epipodial tentacles of *B*. *iridescens* are longer than those of *R*. *eximia*. The lengths of the epipodial tentacles were 2.7, 2.2 and 1.7 mm for *B*. *iridescens* (8.2 mm shell length; from anterior to posterior ones), and 1.7, 1.7, and 1.6 mm for *R*. *eximia* (9.5 mm shell length). These long tentacles may be useful for monitoring the position of their host sea urchin. The presence of opercula varied among Fossarinae species: *F*. *picta* has an operculum which can close up the aperture tightly (Fig 8M and 8Q). In *S*. *pulchella*, however, the operculum is vestigial and too small to close up the aperture (Fig 8N and 8R). In *B*. *iridescens* and *R*. *eximia*, the opercula are completely absent (Fig 8O and 8P). Most gastropods with limpet-shaped shells or loosely coiled auriform shells invariably live clinging to hard substrates, and originally or secondarily lack an operculum [20]. Our results suggest that reduction and loss of the operculum has occurred during the evolution of shell-flattering in Fossarininae.

In Trochidae, Similar depression of shells are seen in Stomatellinae; basal clade *Calliotrochus* has turbiniform shells, while derived clade *Stomatella* has depressed auriform shells [21]. However, there are no species with limpet-shaped shells in Stomatellinae. *Stomatella* snails having strongly flattened shells inhabit undersides of boulders, and their soft bodies are too large to draw them into the shells. These data suggest that the shell flattering in *Stomatella* has occurred as an adaptation to life beneath boulders, and that loss of coiling was not necessary in such protected interstitial habitats.

**Fig 9:**
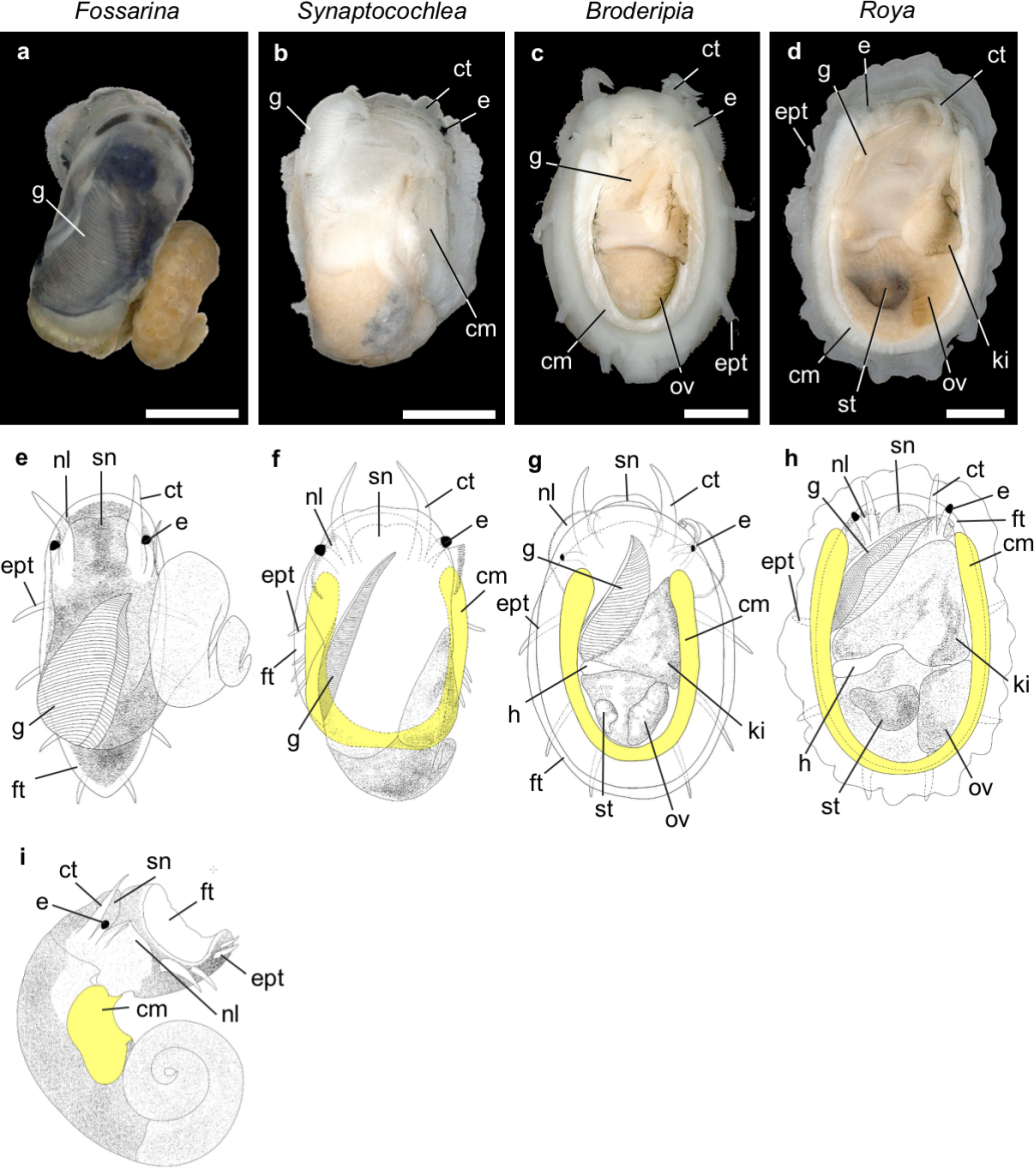
Dorsal views and schematic drawings of soft bodies of four Fossarininae species. *F*. *picta* (a, e, i); *S*. *pulchella* (b, f); *B*. *iridescens* (c, g); *R*. *eximia* (d, h). Columellar muscles in schematic drawings were colored in yellow. *cm* columellar muscle, *ct* cephalic tentacle, *ept* epipodial tentacle, *e* eye, *ft* foot, *g* gill, *h* heart, *ki* kidney, *nl* neck lobe, *ov* ovary, *sn* snout, *st* stomach. *Scale bar* = 1 mm.

The soft bodies of Fossarininae snails corresponded to the shell morphology. In *F*. *picta*, the body is coiled and its anterior is covered by black mantle (Fig 9A and 9E), while anterior parts of the bodies of the other species are whitish and scarcely protruded from the shells. *F*. *picta* has a short, coiled columellar muscle attaching to the columella (Fig 9I). In *S*. *pulchella*, the coiling of the soft body was greatly reduced, and the body is only slightly twisted (Fig 9B). The columellar muscle of *S*. *pulchella* was elongated, horseshoe-shaped and arranged in the posterior part of the body (Fig 9F). In *B*. *iridescens* and *R*. *eximia*, the bodies were vertically contracted, and had completely lost coiling (Fig 9C and 9D), the visceral mass is retracted into the flattened body, and the long, horseshoe-shaped, zygomorphic columellar muscle was situated along the inner margin of the posterior part of the shell. (Fig 9G and 9H). This horseshoe-shaped muscle is suggested to function to appress their shells tightly to the hard substrates.

### Radula morphology and diet of Fossarininae

**Fig 10:**
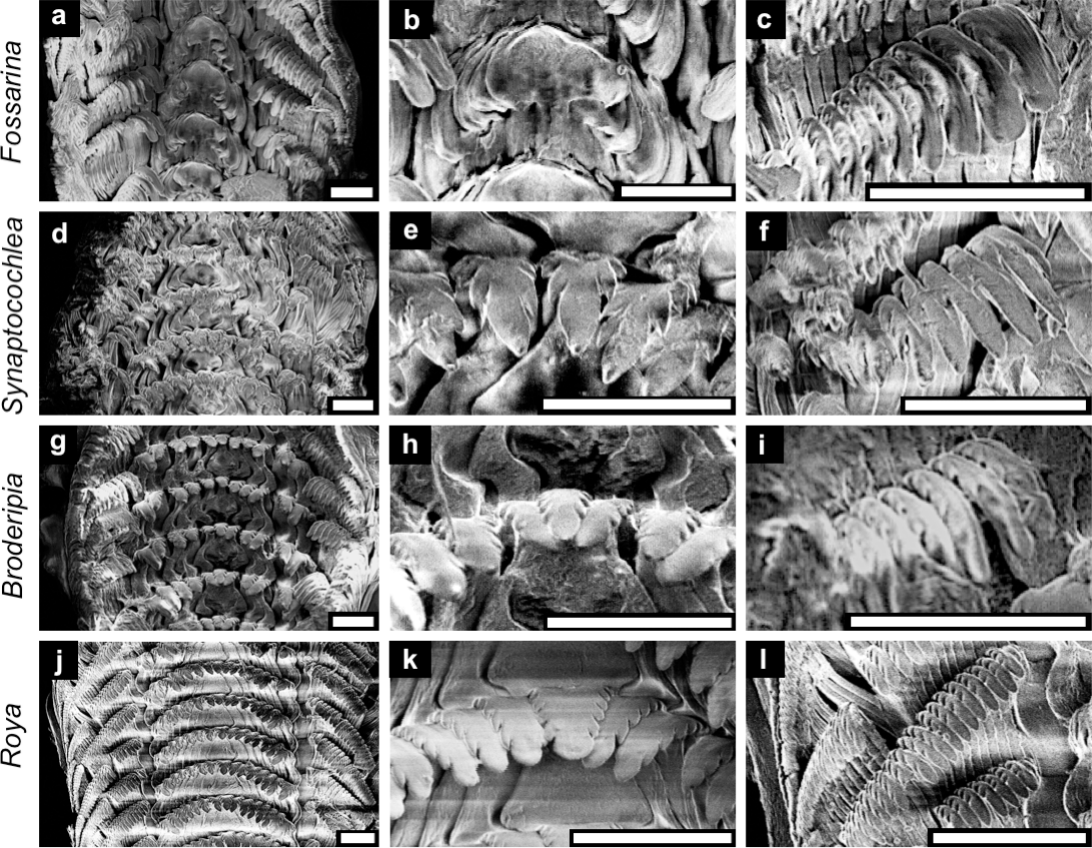
**Views of the whole radulae (left column) and close-up views of the central and marginal teeth (middle and right columns) of four Fossarininae species**. *F*. *picta* (a–c); *S*. *pulchella* (d–f); *B*. *iridescens* (g–i); *R*. *eximia* (j–l). *Scale bar* = 30 m.

Irrespective of shell and soft body morphology differences among the four Fossarininae species, they have largely similar rhipidoglossate radulae. The central and lateral teeth of *F*. *picta* clearly differ from those of the other three species, whereas the marginal teeth were very similar in all four species. The formula of *F*. *picta* radula is (30−40)−4−1−4−(30−40) (Fig 10A). The central tooth has no triangular edge and lies flat along the radula, with the cusp curled slightly upward. The teeth become much narrower in the basal region (Fig 10B). The lateral teeth on each side are hidden by the central tooth or the adjacent lateral tooth. The size and shape of the lateral teeth are similar to those of the central tooth, but the lateral teeth are slightly asymmetric with the outer side being wider than the inner side. The marginal teeth are oar-shaped, with 4−6 serrations on each side (Fig 10C). The formula of *S*. *pulchella* radula is (30−40)−5−1−5−(30−40) (Fig 10D). The central tooth has a sharp triangular edge with 2−3 serrations on each side (Fig 10E). The lateral teeth on each side are similar in size and shape to the central teeth, and bent toward the center of the radula. The marginal teeth are oar-shaped with serrations (Fig 10F). The formula of *B*. *iridescens* radula is (30−40)−5−1−5−(30−40) (Fig 10G). The central tooth has a shovel-shaped triangular edge with 4−6 serrations on each side (Fig 10H). The lateral teeth on each side are similar in size and shape to the central teeth, and are bent toward the center of the radula. The marginal teeth are oar-shaped and serrations are present on all teeth (Fig 10I). The formula of *R*. *eximia* radula is (30−40)−7−1−7−(30−40) (Fig 10J). The shapes and sizes of all teeth of *R*. *eximia* are very similar to those of *B*. *iridescens*, but the cutting edges of the teeth are slightly sharper (Fig 10K and 10L).

As a result of diet examination, diatoms were the primary component of the gastric contents of all four Fossarininae species (Table 3). Fragments of red algae were also observed in small amounts in all snail species, while nematodes and sponge ossicles were rarely observed. The similarity of radulae and diet among Fossarininae species suggest that the differing shell morphologies were not influenced by diet or foraging habits.

**Table 3:** Organic matter seen in the gastric contents of each Fossarininae species.

	*F*. *picta*	*S*. *pulchella*	*B*. *iridescens*	*R*. *eximia*
Pennales diatom	158	19	49	135
red alga	5	2	3	2
Nematoda	0	0	0	1
ossicle of sponge	3	0	2	1
Total	163	21	52	138

### General discussion

**Fig 11:**
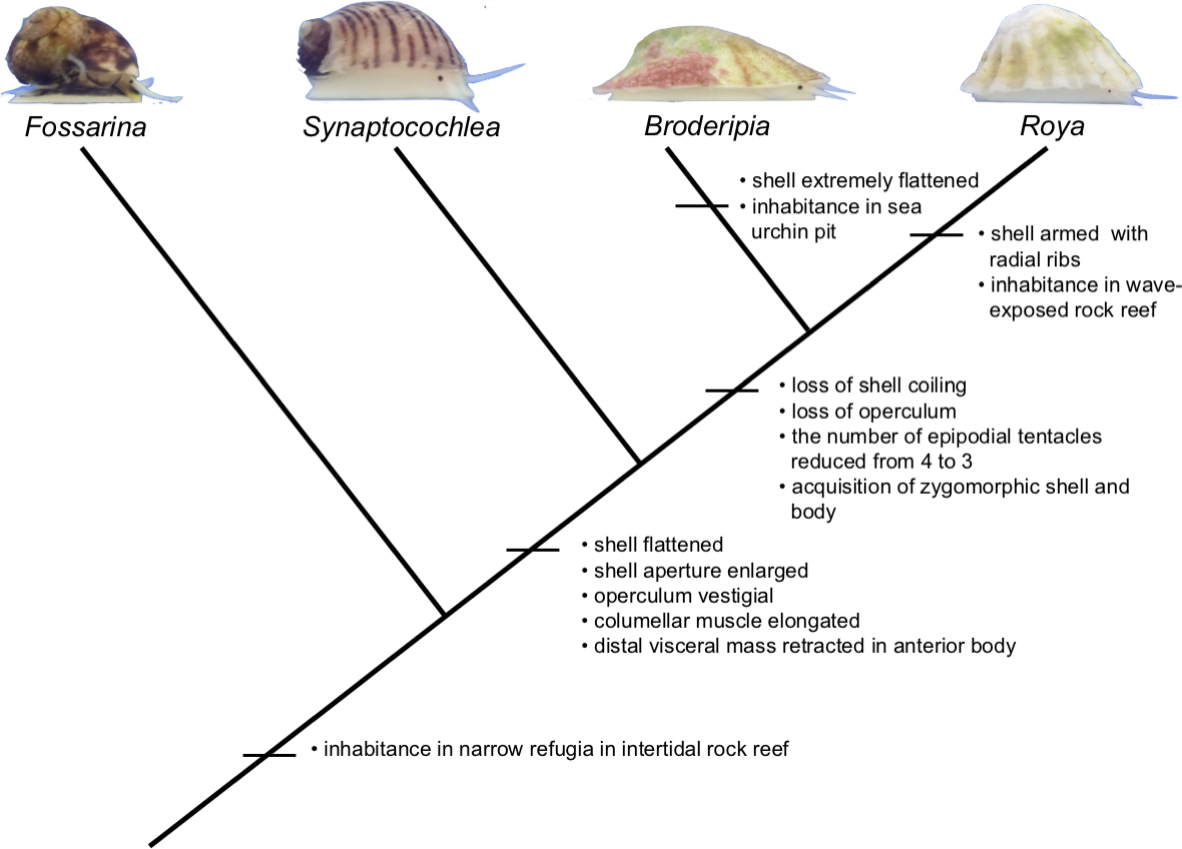
A cladogram of subfamily Fossarininae with the information acquired from this study.

By superimposing the obtained information on morphology and ecology of Fossarininae snails into their cladogram, we can discuss the evolutionary trend accompanying shell-flattering (Fig 11). The basal lineage of the subfamily Fossarininae, *Fossarina*, has a small coiled shell and utilizes narrow refugia in intertidal rock reefs. In *S*. *pulchella*, the coiling of the shell is reduced, aperture is enlarged, and the shell is flattened auriform. *S*. *pulchella* lives in crevices in exposed rock reefs, suggesting that their flat shell with large aperture is adaptive to both the narrow spaces and the wave-swept environment. In the clade comprising *Broderipia* and *Roya*, evolution of a completely zygomorphic limpet-shaped shell has occurred, although selective pressures may differ between the two genera. In *Broderipia*, the extremely flattened limpet-shaped shell is presumed to be an adaptation to the narrow free space offered by the pit of its host sea urchin [7]. In *Roya*, the non-flat limpet-shaped shell with radial ribs is considered to be an adaptation to life moving between wave-swept rock surfaces and refugia of vacant barnacle shells. Our results show that the evolution of limpet-shaped shells occurs under various selective pressures in coiled snail lineages. One intriguing topic for future research is determining the gene whose mutation has contributed to the evolution of limpet-shaped shells in multiple lineages and various marine environments.

## Supporting information

S1 Table Species found in quadrats and degree of coverage of each macroalgal species (n = 10). *SW* strong wave, *GW* gentle wave, *CB* calcareous branched, *CE* calcareous encrusting, *B* branched, *E* encrusting.
(DOCX)
