# Supplementary material for: Morphological and ecological adaptation of limpet-shaped top shells (Gastropoda: Trochidae: Fossarininae) to wave-swept rock reef habitats
Source: PLoS One. 2018 Aug 22;13(8):e0197719. doi: 10.1371/journal.pone.0197719 (PMC6104932; doi:10.1371/journal.pone.0197719)
Supplement: S1 Table — SW strong wave, GW gentle wave, CB calcareous branched, CE calcareous encrusting, B branched, E encrusting. (DOCX) [file pone.0197719.s001.docx]

|  |  |  | Site A | | Site E | |
| --- | --- | --- | --- | --- | --- | --- |
| Phylum | Family | Species | exposed | protected | exposed | protected |
| Rhodophyta | Corallinaceae | *Amphiroa beauvoisii* | 1.06 | 0.88 | 0.00 | 0.00 |
|  |  | *Lithophyllum sp.* | 3.43 | 1.67 | 2.64 | 1.38 |
|  |  | *Serraticardia maxima* | 0.41 | 0.00 | 0.00 | 0.00 |
|  | Gelidiaceae | *Pterocladia capillacea* | 0.06 | 0.05 | 0.44 | 0.69 |
|  | Hildenbrandiaceae | *Hildenbrandia rubra* | 1.96 | 1.67 | 2.78 | 5.85 |
| Ochrophyta | Dermonemataceae | *Dermonema pulvinatum* | 0.00 | 0.00 | 0.59 | 0.00 |
|  | Dictyotaceae | *Dictyopteris divaricata* | 0.01 | 0.00 | 0.00 | 0.00 |
|  |  | *Padina arborescens* | 0.00 | 0.08 | 0.00 | 0.00 |
|  |  | *Spatoglossum pacificum* | 0.00 | 0.01 | 0.00 | 0.00 |
|  | Ishigeaceae | *Ishige okamurae* | 0.00 | 0.00 | 0.73 | 0.00 |
|  | Rhodomelaceae | *Palisada intermedia* | 0.08 | 0.77 | 0.39 | 0.00 |
|  | Sargassaceae | *Sargassum fusiforme* | 1.14 | 0.00 | 0.00 | 0.00 |
|  |  | *Sargassum patens* | 0.97 | 0.15 | 0.00 | 0.00 |
|  |  | bare rock | 0.80 | 2.26 | 1.26 | 1.97 |
|  |  | sand | 0.00 | 2.43 | 0.00 | 0.00 |
| Arthropoda | Balanidae | *Fistulobalanus albicostatus* | 0.00 | 0.00 | 0.10 | 4.20 |
|  |  | *Megabalanus volcano* | 1.70 | 0.00 | 0.00 | 0.00 |
|  | Tetraclitidae | *Tetraclita japonica* | 0.10 | 0.00 | 0.60 | 0.00 |
|  |  | *Tetraclita squamosa* | 0.30 | 0.00 | 8.30 | 0.00 |
|  | Chthamalidae | *Chthamalus challengeri* | 0.00 | 0.00 | 0.00 | 4.20 |
|  | Caprellidae | *Caprella penantis* | 0.30 | 0.00 | 0.00 | 0.00 |
|  | Hyalidae | *Hyale sp.* | 0.10 | 0.00 | 0.00 | 0.00 |
|  | Maeridae | *Elasmopus* sp. | 4.10 | 0.00 | 0.00 | 0.00 |
|  | Majidae | *Tiarinia cornigera* | 0.10 | 0.00 | 0.00 | 0.00 |
|  | Paguridae | *Pagurus filholi* | 0.00 | 0.30 | 0.00 | 0.00 |
|  | Sphaeromatidae | *Dynoides dentisinus* | 0.30 | 0.00 | 0.10 | 0.30 |
|  |  | *Tanaidacea* sp. | 0.00 | 0.00 | 0.10 | 0.00 |
| Annelida | Spirorbidae | *Janua foraminosa* | 0.00 | 2.30 | 0.00 | 0.00 |
| Mollusca | Acanthochitonidae | *Acanthochitona achates* | 0.00 | 0.00 | 0.20 | 0.00 |
|  | Arcidae | *Arca avellana* | 0.00 | 0.20 | 0.00 | 0.00 |
|  | Calyptraeidae | *Crepidula gravispinosus* | 0.40 | 0.00 | 0.00 | 0.00 |
|  | Chitonidae | *Rhyssoplax kurodai* | 0.60 | 0.00 | 0.00 | 0.10 |
|  | Fissurellidae | *Diodora quadriradiata* | 0.10 | 0.00 | 0.00 | 0.00 |
|  |  | *Tugali decussata* | 0.20 | 0.00 | 0.00 | 0.00 |
|  |  | *Montfortula picta* | 1.30 | 0.00 | 0.10 | 0.00 |
|  | Haminoeidae | *Haloa japonica* | 0.00 | 0.00 | 0.10 | 0.00 |
|  | Haliotidae | *Haliotis varia* | 0.80 | 0.00 | 0.00 | 0.00 |
|  | Hipponicidae | *Antisabia foliacea* | 0.00 | 0.10 | 0.00 | 0.00 |
|  |  | *Sabia conica* | 0.00 | 0.10 | 0.00 | 0.00 |
|  | Lottiidae | *Collisella langfordi* | 2.10 | 0.00 | 2.30 | 0.40 |
|  |  | *Lottia dorsuosa* | 0.00 | 0.00 | 0.20 | 0.00 |
|  |  | *Lottia kogamogai* | 0.10 | 0.00 | 0.10 | 0.30 |
|  |  | *Lottia tenuisculpta* | 0.90 | 0.00 | 0.50 | 0.00 |
|  |  | *Patelloida saccharina* | 0.00 | 0.00 | 0.90 | 0.00 |
|  | Mopaliidae | *Placiphorella stimpsoni* | 0.10 | 0.00 | 0.00 | 0.00 |
|  | Muricidae | *Cronia margariticola* | 0.00 | 0.50 | 0.00 | 0.00 |
|  |  | *Maculotriton serriale* | 0.10 | 0.00 | 0.00 | 0.00 |
|  |  | *Morula granulata* | 0.10 | 0.00 | 0.00 | 0.00 |
|  |  | *Morula iostoma* | 0.20 | 0.00 | 0.00 | 0.00 |
|  |  | *Morula musiva* | 0.00 | 0.40 | 0.00 | 0.00 |
|  |  | *Thais clavigera* | 0.00 | 0.10 | 0.00 | 0.00 |
|  |  | *Thais luteostoma* | 0.00 | 0.00 | 0.00 | 0.30 |
|  | Mytilidae | *Lithophaga curta* | 0.10 | 0.00 | 0.00 | 0.00 |
|  |  | *Modiolus nipponicus* | 0.10 | 0.00 | 0.00 | 0.00 |
|  | Nacellidae | *Cellana toreuma* | 0.00 | 0.10 | 2.00 | 2.50 |
|  | Ostreidae | *Saccostrea kegaki* | 0.00 | 0.00 | 0.00 | 0.10 |
|  | Patellidae | *Scutellastra flexuosa* | 0.10 | 0.00 | 0.20 | 0.10 |
|  | Siphonarioiidae | *Siphonaria sirius* | 0.00 | 0.30 | 1.50 | 0.90 |
|  |  | *Siphonaria japonica* | 0.00 | 0.00 | 0.70 | 1.20 |
|  | Tegulidae | *Chlorostoma turbinatum* | 0.00 | 0.00 | 0.00 | 0.10 |
|  | Trochidae | *Broderipia iridescens* | 3.90 | 0.00 | 0.00 | 0.00 |
|  |  | *Fossarina picta* | 0.50 | 0.60 | 0.00 | 0.00 |
|  |  | *Roya eximia* | 0.10 | 0.00 | 2.20 | 0.00 |
|  |  | *Synaptocochlea pulchella* | 0.20 | 0.00 | 0.00 | 0.00 |
|  |  | *Pictodiloma suavis* | 0.00 | 0.00 | 0.60 | 0.10 |
|  | Vermetidae | *Serpulorbis imbricatus* | 0.10 | 0.10 | 0.10 | 0.30 |
| Echinodermata | Echinometridae | *Anthocidaris crassispina* | 0.70 | 0.10 | 0.00 | 0.00 |
|  |  | *Echinostrephus* spp. | 0.70 | 0.50 | 0.00 | 0.00 |
|  | Toxopneustidae | *Toxopneustes pileolus* | 0.10 | 0.00 | 0.00 | 0.00 |
| Chordata | Chaenopsidae | *Neoclinus bryope* | 0.00 | 0.10 | 0.00 | 0.00 |
|  | Blenniidae | *Rhabdoblennius nitidus* | 0.00 | 0.10 | 0.00 | 0.00 |
